# Integrative Analysis of Metabolome and Transcriptome Provides Insights into the Mechanism of Flower Induction in Pineapple (*Ananas comosus* (L.) Merr.) by Ethephon

**DOI:** 10.3390/ijms242417133

**Published:** 2023-12-05

**Authors:** Wenqiu Lin, Shenghui Liu, Xiou Xiao, Weisheng Sun, Xinhua Lu, Yuyao Gao, Junjun He, Zhuying Zhu, Qingsong Wu, Xiumei Zhang

**Affiliations:** 1 South Subtropical Crop Research Institute, Chinese Academy of Tropical Agricultural Sciences, Zhanjiang 524091, China; linwenqiu1989@163.com (W.L.);; 2 Laboratory of Tropical Fruit Biology, Ministry of Agriculture, Zhanjiang 524091, China; 3 Key Laboratory of Hainan Province for Postharvest Physiology and Technology of Tropical Horticultural Products, Academy of Tropical Agricultural Sciences, Zhanjiang 524091, China

**Keywords:** pineapple, flower induction, metabolome, transcriptome, regulatory network

## Abstract

Exogenous ethylene is commonly utilized to initiate flower induction in pineapple (*Ananas comosus* (L.) Merr.). However, the molecular mechanisms and metabolic changes involved are not well understood. In this study, we explored the genetic network and metabolic shifts in the ‘Comte de Paris’ pineapple variety during ethylene-induced flowering. This was achieved through an integrative analysis of metabolome and transcriptome profiles at vegetative shoot apexes (0 d after ethephon treatment named BL_0d), the stage of bract primordia (8 d after ethephon treatment named BL_8d), stage of flower primordia (18 d after ethephon treatment named BL_18d), and the stage of stopped floret differentiation (34 d after ethephon treatment named BL_34d). We isolated and identified 804 metabolites in the pineapple shoot apex and inflorescence, categorized into 24 classes. Notably, 29, 31, and 46 metabolites showed significant changes from BL_0d to BL_8d, BL_8d to BL_18d, and BL_18d to BL_34d, respectively. A marked decrease in indole was observed, suggesting its role as a characteristic metabolite during flower induction. Transcriptomic analysis revealed 956, 1768, and 4483 differentially expressed genes (DEGs) for BL_0d vs. BL_8d, BL_8d vs. BL_18d, and BL_18d vs. BL_34d, respectively. These DEGs were significantly enriched in carbohydrate metabolism and hormone signaling pathways, indicating their potential involvement in flower induction. Integrating metabolomic and transcriptomic data, we identified several candidate genes, such as *Agamous-Like9* (*AGL9*), *Ethylene Insensitive 3-like* (*ETIL3*), *Apetala2* (*AP2*), *AP2-like ethylene-responsive transcription factor ANT* (*ANT*), and *Sucrose synthase 2* (*SS2*), that play potentially crucial roles in ethylene-induced flower induction in pineapple. We also established a regulatory network for pineapple flower induction, correlating metabolites and DEGs, based on the *Arabidopsis thaliana* pathway as a reference. Overall, our findings offer a deeper understanding of the metabolomic and molecular mechanisms driving pineapple flowering.

## 1. Introduction

Flowering represents a fundamental physiological process in plants that is essential for genetic continuity and holds particular significance in agriculture. The transition to flowering, a pivotal phase in the plant life cycle, is tightly regulated by complex networks that involve multiple environmental and internal signals ensuring the precise timing of this crucial event [1,2]. Key genes implicated in the regulation of flowering, including *FLOWER LOCUS T* (*FT*) [3], *LEAFY* (*LFY*) [4], *APETALA2* (*AP2*) [5], and *SQUAMOSA PROMOTER BINDING PROTEIN–LIKE* (*SPL*) [6] have been identified as pivotal players in controlling flowering time in plants. These genes predominantly align with six major flowering pathways: aging, autonomous, gibberellic acid (GA), photoperiod, thermosensory, and vernalization pathways. Notably, recent advancements have unveiled the integration of carbohydrates into these known flowering pathways [7,8], indicating their synergistic contribution to regulating flowering time.

Carbohydrates play dual roles in flowering, acting both as signaling molecules and energy sources during this critical process [9]. In *Arabidopsis thaliana*, there is a notable surge in sucrose content in the apical regions during flower induction. Further studies have shown that low concentrations of sucrose promote flowering, while higher concentrations exert inhibitory effects [10]. In the case of *Juglans sigillata*, sugar emerges as an early signaling molecule for floral induction, particularly in girdling-mediated floral induction [11]. Sugar derivatives have also demonstrated their involvement in flowering, regulation, exemplified by the upsurge in *trehalose-6-phosphate* (*T6P*) in the meristems of wild-type *Arabidopsis thaliana* plants, and lower levels of *TREHALOSE-PHOSPHATE SYNTHASE 1* (*TPS1*) down-regulated *FT*, resulting in a substantial delay in flowering [7]. Additionally, studies have elucidated the role of carbon, lipids, and fatty acid derivatives in the induction of flowering [9].

Plant hormones exert a significant influence on plant development and the timing of flowering. Ethylene (ET), indole-3-acetic acid (IAA), cytokinins (CKs), and abscisic acid (ABA) [12,13] act as key regulators of flower induction through the GA [14], autonomous [15], and photoperiod [16] pathways. The application of exogenous ethylene (ethephon) has been widely used to induce pineapple flowering, enhancing the release of endogenous ethylene to stimulate this process [17]. However, ethylene can also inhibit flowering by elevating the levels of bioactive gibberellins (GA) and promoting the accumulation of DELLA proteins, consequently downregulating the expression of *AP1*, *LEAFY*, and *SOC1* [18]. Exogenous cytokinin (CK) application has been shown to promote flower induction by modulating the expression of flowering-related genes such as *FT* and *SOC1* [19]. IAA plays a crucial role in flowering regulation with *IAA/AUX* binding to the promoters of *APETALA1* (*AP1*) and *FRUITFULL* (*FUL*) in woodland strawberry [20]. Abscisic acid (ABA), on the other hand, influences flowering through the photoperiodic and sugar signaling pathways [21,22]. Furthermore, additional plant hormones, including jasmonic acid (JA) and salicylic acid (SA), contribute to flowering regulation and participate in multiple biological processes [23,24].

Pineapple (*Ananas comosus* (L.) Merr.) is one of the most important tropical fruits in the world. However, the natural flowering of pineapple is subject to various factors, such as variety, plant size, plant age, shortened day length, cool night temperatures, and growing conditions, leading to disparities in flowering timing and rates [17,25]. Natural flowering can extend harvest time and even result in the production of undersized, unmarketable fruits [26]. To address this challenge, ethephon, an ethylene-releasing compound, is commonly used to induce pineapple flowering. Ethephon effectively fine-tunes flowering timing, elevates flowering rates, and synchronizes the flowering [27]. Exogenous ethylene enhances endogenous ethylene release and reduces GA levels, thus facilitating pineapple flowering [17]. Despite the pivotal role of metabolites in pineapple flowering, they have remained relatively understudied with only a few investigations conducted. In pursuit of a deeper understanding of the mechanisms underpinning flower induction by ethephon, the cloning of aminocyclopropane carboxylic acid synthase genes (*ACC*) has been achieved. Subsequent research has shown that silencing the *AcACS2* gene can lead to a delay in flowering time in transgenic plants [28]. Moreover, *ERF* and *RAV* subfamily genes are likely to play crucial roles in ethylene-induced pineapple flowering [29,30], although the complex network governing pineapple flowering remains to be fully elucidated.

In this study, we conducted an in-depth investigation into the transcriptomic and metabolic dynamics of shoot apexes and inflorescences to elucidate the molecular mechanisms and metabolic constituents that underlie the process of pineapple flowering. These findings contribute significantly to a comprehensive understanding of the dynamic alterations in metabolites and the molecular regulatory mechanisms governing pineapple flowering.

## 2. Results

### 2.1. Overview of Metabolite Profiles during Flower Induction

**Figure 1:**
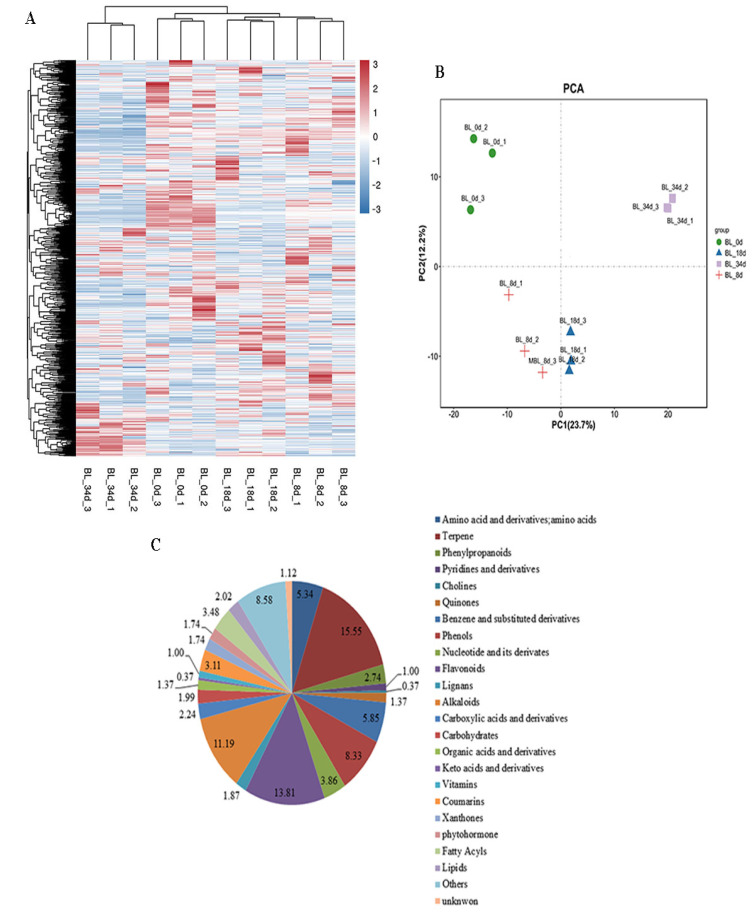
Qualitative and quantitative analysis of the metabolomics data of shoot apexes or inflorescences in pineapple. (**A**) Heatmap of metabolites. The color scale indicates abundance of metabolites. Darker red colors indicate higher metabolite abundance, while lighter blue color indicate lower metabolite abundance. (**B**) PCA analysis of shoot apexes or inflorescences. (**C**) Component analysis of the identified metabolites from shoot apexes or inflorescences.

To gain deeper insights into the metabolomics profiling of relevant compounds during flower induction by ethephon, we identified primary and secondary metabolites within the stem apexes and inflorescences of pineapple by UPLC-MS/MS analysis (Table S1). A total of 804 distinct annotated metabolites were discerned across different flowering buds, categorized into 24 distinct classes. Clustering based on metabolite abundance resulted in the grouping of 12 samples into four distinct clusters, with significant correlations observed among samples within the same cluster (Figure 1A and Figure S1). Furthermore, principal component analysis (PCA) showed that the first two principal components (PCs) explained 35.9% of the total variance (PC1 = 23.7%, PC2 = 12.2%), showing distinct variations in metabolites within flowering buds during the process of flower induction (Figure 1B).

Among the identified metabolites, we identified 43 amino acids and derivatives (5.34%), 125 terpenes (15.55%), 22 phenylpropanoids (2.74%), 8 pyridines and derivatives (1.00%), 3 cholines (0.37%), 11 quinones (1.37%), 47 benzene and substituted derivatives (5.85%), 67 phenols (8.33%), 31 nucleotides and their derivates (3.86%), 111 flavonoids (13.81%), 15 lignans (1.87), 90 alkaloids (11.19%), 18 carboxylic acids and derivatives (2.24%), 16 carbohydrates (1.99%), 11 organic acids and derivatives (1.37%), 3 keto acids and derivatives (0.37%), 8 vitamins (1.00%), 25 coumarins (3.11%), 14 xanthones (1.74%), 14 phytohormones (1.74%), 28 fatty acyls (3.48%), 16 lipids (2.02%), and 69 other metabolites (8.58%). Additionally, 9 metabolites remained unidentified (1.12%) (Figure 1C). A total of 799, 798, 799, and 795 metabolites were detected from BL_0d, BL_8d, BL_18d, and BL_34d, respectively (Table S1).

**Figure 2:**
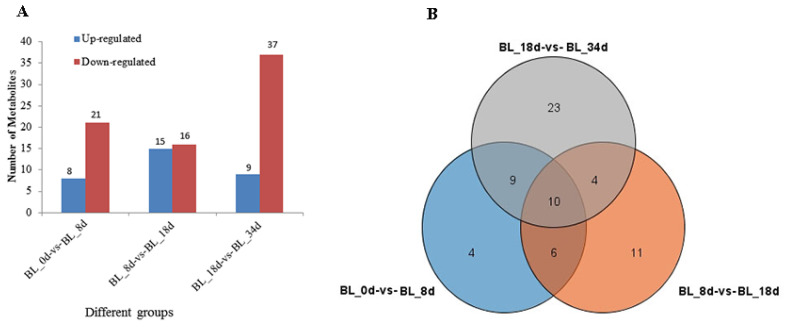
The analysis of differentially accumulated metabolites (DAMs). (**A**) Histogram of DAMs; (**B**) Venn diagram of DAMs.

Differential analysis using variable importance in projection (VIP) with a threshold of ≥1 and *t*-test *p* < 0.05 revealed differentially accumulated metabolites (DAMs). Between BL_0d and BL_8d, we detected 29 DAMs comprising 8 up-regulated and 21 down-regulated metabolites. The transition from BL_8d to BL_18d exhibited 31 DAMs with 15 up-regulated and 16 down-regulated metabolites. Finally, between BL_18d and BL_34d, 46 DAMs were identified, with 9 up-regulated and 37 down-regulated metabolites, respectively (Figure 2A). Up-regulated metabolites included acidic amino acids and carbohydrates including glutamic acid, aspartic acid, and stachyose. Conversely, most of the DAMs related to plant hormones, organic acids, and carbohydrates were down-regulated, including indole, cis-aconitic acid, 1-kestose, and maltotriose (Figure S2). Further scrutiny of consistently altered metabolites during flower induction revealed 10 metabolites, including glutamine, diallyl disulfide, and indole, which serve as distinctive markers of flowering induction (Figure 2B and Figure S3).

### 2.2. Transcriptome Analyses

**Figure 3:**
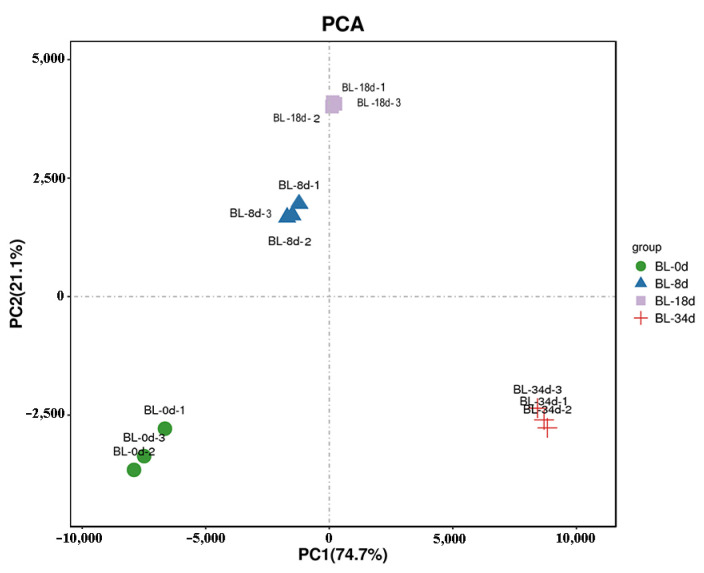
PCA analyses of shoot apexes or inflorescences in pineapple.

To investigate the gene expression profiles associated with flower induction through ethephon treatments, RNA-seq analysis was performed at four time points: 0 d, 8 d, 18 d, and 34 d. The clean data obtained for each sample ranged from 40,693,802 to 54,427,760 after filtering out low-quality raw data. Reads aligned to ribosomal sequences were excluded to derive the total reads, which were subsequently mapped to the pineapple genome (Ensembl v48), achieving mapping rates ranging from 74.02% to 85.72% (Table S2). Principal component analysis (PCA) revealed high similarity among the three biological replicates (Figure 3), while clear separation of samples from different stages underscored the robust repeatability of our results (Figure S4).

**Figure 4:**
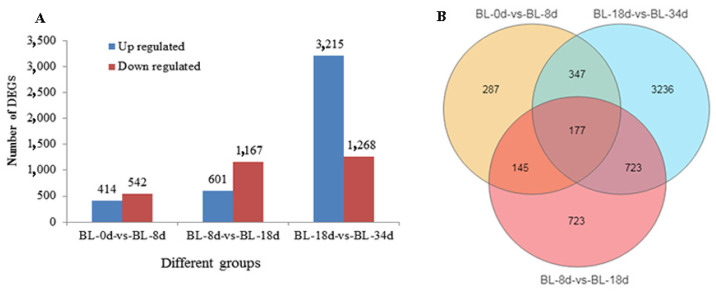
Differentially expressed genes (DEGs) of pineapple shoot apexes or inflorescences. (**A**) The numbers of up- and down-regulated genes for BL_0d vs. BL_8d, BL_8d vs. BL_18d, and BL_18d vs. BL_34d; (**B**) Venn diagram showing the number of overlapping DEGs in flowering induction.

Further examination of gene expression profiles was conducted by calculating FPKM (fragments per kilobase of transcript per million fragments mapped) values for each sample. Differential expression analyses showed that a total of 956, 1768, and 4483 DEGs were detected in the BL_0d vs. BL_8d, BL_8d vs. BL_18d, and BL_18d vs. BL_34d comparisons, respectively. Notably, the number of differentially expressed genes (DEGs) increased as flowering progressed. Specifically, when compared to the 0 d time point, we observed 414 up-regulated genes and 542 down-regulated genes at 8 d. The transition to 18 d resulted in 601 up-regulated genes and 1167 down-regulated genes in contrast to 8 d. Finally, in comparison to 18 d, 3215 up-regulated genes and 1268 down-regulated genes were identified at 34 d (Figure 4A). Venn diagram analysis suggested that 177 DEGs were consistently altered across all three time points (Figure 4B).

### 2.3. Functional Annotation of Differentially Expressed Genes

**Figure 5:**
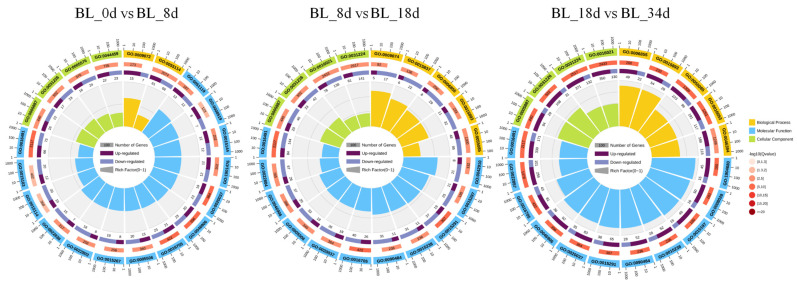
GO enrichment circular plots of DEGs. The top 20 GO terms were enriched in the first circle with the coordinate ruler of the number of genes noted outside the outermost circle. Different colors represent different ontologies. The numbers and the Q value of GO terms are presented in differential gene background in the second circle. The more background genes were calculated, the longer the bars are. Lower Q values are indicated by darker red colors. The ratio of upregulated and downregulated differential genes is shown with a bar graph in the third circle, where dark and light purple represent the ratios of upregulated and downregulated genes, respectively; specific values are displayed below the bars. The Rich Factor value of each GO term is displayed in the fourth (innermost) circle (the number of differentially expressed genes in the pathway divided by all genes).

**Figure 6:**
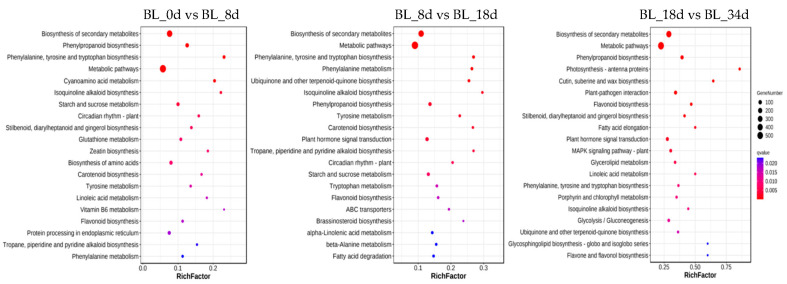
KEGG enrichment of DEGs for three comparison groups (BL_0d vs. BL_8d, BL_8d vs. BL_18d, BL_18d vs. BL_34d). Each bubble represents a pathway where abscissa and bubble size together indicate the magnitude of the impact factors of the pathway.

In this study, we conducted a comprehensive analysis of DEGs in pineapple flower induction, employing both Gene Ontology (GO) and Kyoto Encyclopedia of Genes and Genomes (KEGG) enrichment analyses to elucidate the biological functions underlying this process. Our GO annotation highlighted the enrichment of DEGs in various processes, including ‘aromatic amino acid family metabolic process (GO: 0009072)’, ‘carbohydrate transporter activity (GO: 1901476)’, ‘transmembrane transport (GO:0055085)’, ‘oxidoreductase activity (GO:0016491)’, and ‘component of membrane (GO:0005887 and GO:0031226)’ (Figure 5, Table S3). Furthermore, the DEGs were significantly enriched in KEGG pathways such as ‘Biosynthesis of secondary metabolites (ko01110)’ and ‘Metabolic pathways (ko01100)’, including ‘Starch and sucrose metabolism (ko00500)’, ‘Circadian rhythm-plant (ko04712)’ and ‘Plant hormone signal transduction (ko04075)’ (Figure 6). These results indicate that carbohydrate transporter and plant hormone signal transduction might have an important role in the induction of pineapple flowers.

### 2.4. Integrative Analysis of DAMs and DEGs

**Figure 7:**
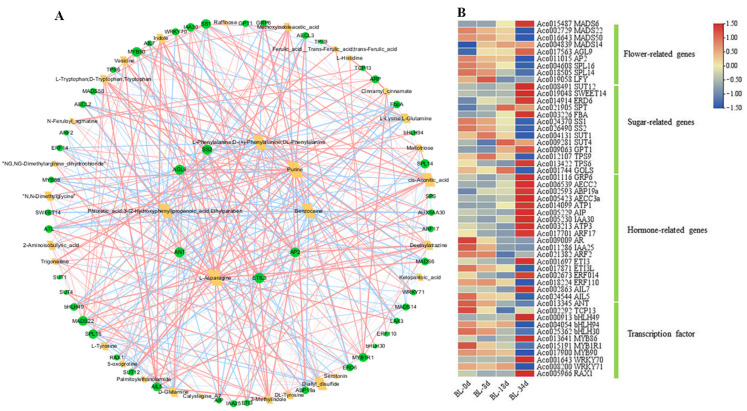
The analyses of correlation network of DEGs and DAMs. (**A**) Network of DEGs and DAMs by more than 0.9 relationship pairs of correlation coefficient absolute value. Green dots represent genes and yellow quadrangles represent metabolites. The size of dots indicates connectivity in the network. The red lines show positive correlations of related pairs and blue lines show negative correlations. (**B**) Heat map of DEGs using RNA-seq data.

To gain a deeper understanding of the relationship between metabolites and genes during flowering induction, we conducted a correlation analysis between differentially accumulated metabolites (DAMs) and differentially expressed genes (DEGs), with a particular focus on those related to carbohydrates, plant hormones, and flowering induction. This analysis resulted in a correlation network comprising 48 genes and 32 metabolites, including a total of 82 nodes and 392 edges (Figure 7A). Noteworthy findings from this network analysis include 9 flowering-related genes (3 upregulated and 6 downregulated), 13 sugar-related genes (8 upregulated and 5 downregulated), 18 hormone-related genes (12 upregulated and 6 downregulated), and 10 transcription factors (3 upregulated and 7 downregulated) (Figure 7B). Notably, genes such as *AGL9* (Aco017563), *ETIL3* (Aco017871), *AP2* (Aco011015), *ANT* (Aco013345), and *SS2* (Aco026490) occupied pivotal positions within the network (Figure 7A), suggesting their potential candidacy in the regulation of shoot apexes or inflorescences following ethephon treatment.

**Figure 8:**
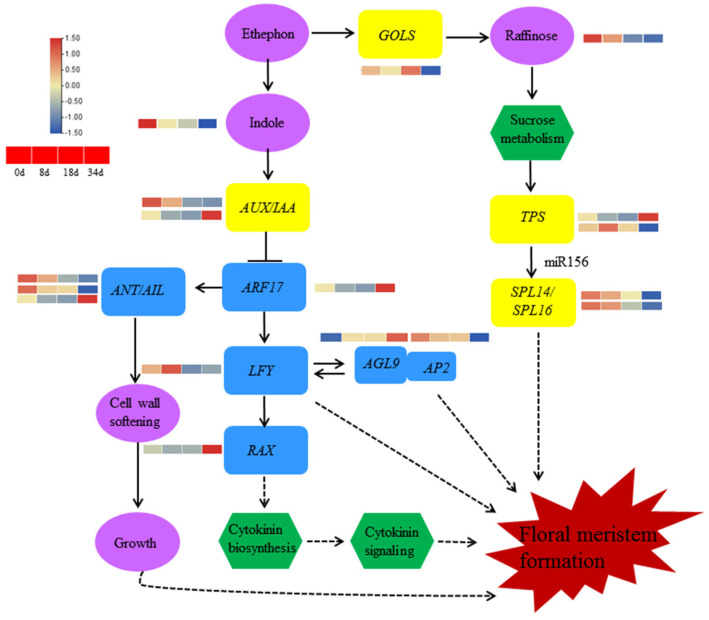
Genetic network involved in pineapple floral meristem formation. This model is based on the publications by Denay et al. [31] and Chakraborty et al. [11].

Sugars and hormones have well-established roles in floral meristem formation. To further investigate the regulatory network governing pineapple flowering, we constructed a map of genes and metabolites based on the reference of floral meristem formation in *Arabidopsis thaliana* (Figure 8). In this context, we identified indole and raffinose from an integrative analysis of DAMs and DEGs, and amounts of indole and raffinose were decreased. This observation implies potential roles for indole signaling, cytokinin signaling, or carbohydrate signaling pathways in the regulation of pineapple flowering. Additionally, we identified three DEGs associated with auxin response factors. Among them, two *AUX/IAA* genes showed differential expression, with one upregulated and one downregulated, and one was an *ARF17* gene exhibiting upregulation during pineapple floral meristem formation. Furthermore, the transcriptional levels of *GOLS* and *TPS9*, which are related to carbohydrate metabolism, were upregulated, while *TPS7* was downregulated. The transcription factors *ANT* and *AIL5* showed upregulation, whereas *AIL7* was downregulated during floral outgrowth. Five genes were connected to flowering formation, including *AGL9* (upregulated), *LFY*, *AP2*, *SPL14*, and *SPL16* (all downregulated) (Figure 8).

### 2.5. Verification of DEGs by qPCR

**Figure 9:**
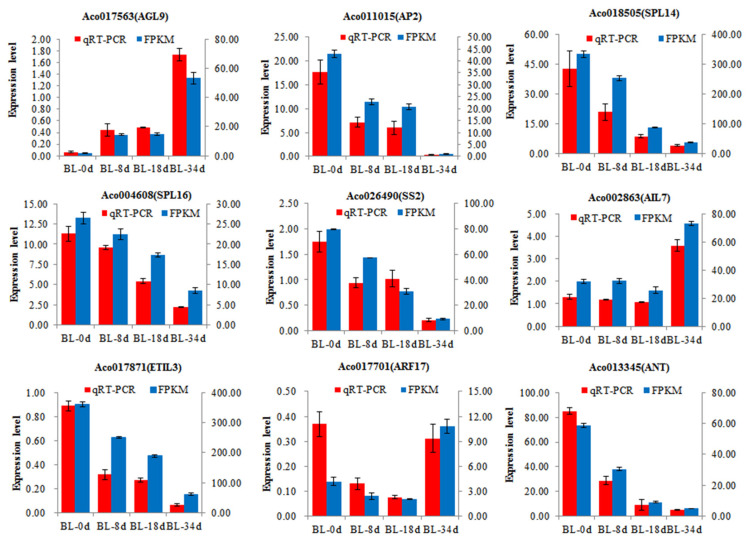
Validation of 9 DEGs identified during flowering induced using RNA-seq and qRT-PCR. Red bars represent qRT-PCR (log10^2−ΔΔCT^) and blue bars represent RNA-seq (log_2_FoldChange). Error bars show standard deviation of three independent duplicates.

To validate our transcriptome results, a total of 9 genes, which included 5 potential candidate genes *AGL9* (Aco017563), *ETIL3* (Aco017871), *AP2* (Aco011015), *ANT* (Aco013345), and *SS2* (Aco026490), were selected for qRT-PCR analysis. These genes were related to flowering pathway (Aco017563, Aco011015, Aco018505, and Aco004608), carbohydrate metabolism (Aco026490), plant hormones (Aco002863, Aco017871, and Aco017701), and transcription factors (Aco013345). The qRT-PCR data corroborated the expression trends observed in RNA sequencing results, thus confirming the reliability of the RNA-seq analysis (Figure 9).

## 3. Discussion

Natural flowering leads to a lack of desynchronization in fruit harvest disrupting fruiting schedules [26]. To address this issue and ensure uniformity in harvest timing, various regulators have been used to induce flowering in pineapple [32]. Ethephon is widely applied for flower induction due to the effective rate and uniformity of pineapple [17,26]. Additionally, studies have highlighted changes in sugar and plant hormone content during inflorescence induction, suggesting their involvement in flower induction [17]. However, it remains unclear if other metabolites also participate in this process. In our study, we identified a total of 804 metabolites through metabolome analysis, categorizing them into 24 classes including amino acids and derivatives, terpenes, phenylpropanoids, pyridines and derivatives, cholines, quinones, benzene and substituted derivatives, phenols, nucleotides and their derivatives, flavonoids, lignans, alkaloids, carboxylic acids and derivatives, carbohydrates, organic acids and derivatives, keto acids and derivatives, vitamins, coumarins, xanthones, phytohormones, fatty acyls, and lipids (Figure 1C). These findings provide insights into the metabolic profiles during pineapple flower induction. Furthermore, our study identified at least 29 DAMs during flower induction, with 10 of these metabolites demonstrating either an increase or decrease in abundance with the development of flowering (Figure S3). These metabolites may serve as potential biomarkers for flower induction.

Hormone regulation and signaling plays a critical role in the induction of flowering [33,34,35]. Ethylene, for instance, has been shown to exert a delaying effect on flowering time, impeding the transition from vegetative to reproductive growth in *Arabidopsis* and *Chrysanthemum*, while promoting flowering in pineapple [36,37]. Several reports have elucidated the regulatory role of the *AP2/ERF* family in modulating flowering time [37,38]. For example, in *Arabidopsis*, overexpression of *AtERF1* significantly delays the flowering time process, whereas *ERF1* knockout expedites it [37]. In contrast, in *Chrysanthemum*, overexpression of *CmERF110* results in earlier flowering compared to the wild-type, while the suppression of *ERF110* leads to delayed flowering [38]. Pineapple, with its 97 identified members of the *AP2/ERF* family, potentially plays a pivotal role in responding to ethylene [30]. In our study, we observed that following ethephon treatment, *ERF014* exhibited upregulated expression, whereas *ERF110* displayed downregulated expression, indicating distinct functions of ERFs in response to ethylene-mediated flowering regulation (Figure 7B). It has been documented that exogenous ethylene elevates endogenous ethylene levels while decreasing indole-3-acetic acid content, thereby inducing flowering in pineapple [17]. In our investigation, a significant decrease in indole levels was noted, underscoring its crucial role in flower induction.

The initiation of floral primordia necessitates a local auxin maximum and *auxin response factor5* (*ARF5*) activity [39]. *AIL6* stands out as a key regulator of floral primordium initiation, targeting *ARF* for transcriptional activation [40]. Overexpression of *TaAUX/IAA15-1A* in *Brachypodium* results in early flowering time through its interaction with *ARF* [41]. In our study, we found that the transcript levels of *AUX/IAA25* and *AIL5* decreased during flower induction, whereas the transcript level of *ARF17* increased, indicating a distinctive regulatory role for indole in plant flowering [26]. Additionally, the transcript levels of *AUX/IAA30* and *AIL7* increased, displaying a contrary pattern to that observed in *AUX/IAA25* and *AIL5* (Figure 7B). Although we did not detect differentially abundant metabolites of ethylene and its derivatives during pineapple flower induction, variations in the transcript levels of ethylene-related genes such as *ERF110*, *ERF014*, and *ETI3* were evident, suggesting potential interactions between ethylene-related genes and other flowering pathways (Figure 7B). Overall, our study provides insights into the roles of hormone regulation and signaling, specifically ethylene and indole, in pineapple flower induction. Further research is imperative to gain a comprehensive understanding of the integration of these pathways in regulating flowering.

Carbohydrates and their derivatives serve not only as primary energy sources but also as signaling molecules in flower induction [42,43]. In the context of pineapple flower induction, sucrose levels exhibited an increase within 24 h after ethephon treatment [26]. The transcripts of *SS1*, *SS2*, *SUT1*, and *TPS9* shown an increase (Figure 7B), which was similar to the expression patterns observed in the lily hybrid ‘Sorbonne’ [44]. Further analysis in *Arabidopsis thaliana* revealed that the overexpression of *LoSUT2* and *LoSUT4* resulted in earlier blooming. *SUT* was shown to directly regulate SS genes involved in carbohydrate metabolism downstream [44]. *Trehalose-6-phosphate* (*T6P*) emerged as a pivotal signaling molecule in sugar metabolite processes, and *Trehalose-6-phosphate synthase* (*TPS*) catalyzed the production of T6P, playing a crucial role in flower induction and development [7,45]. In apple, sucrose and T6P levels were high in the early stages of flower induction and gradually decreased, while *TPS7* expression increased during flower induction, indicating the significant involvement of *T6P* in initiating flower induction [46]. Conversely, *TPS6*, *SWEET14*, *SUT4*, and *SUT12* expression decreased, indicating the complexity and rigor of the carbohydrate pathway (Figure 9). This evidence suggests that these genes may be involved in the carbohydrate signaling pathway that regulates flowering. Furthermore, *GOLS* transcript levels decreased during flower induction, accompanied by a decrease in raffinose levels (Figure 8), highlighting the participation of *GOLS* in flowering by its response to sugar regulation.

The identification of differential abundant metabolites and correlation analysis with DEGs has provided valuable insights into the mechanisms of flower induction [20,46]. In *Prunus mume*, 18 DEGs were identified forming a correlation network that highlights four key genes [20]. In this study, 48 genes were screened for their relevance between differentially abundant metabolites and differential genes. Among them, *AGL9*, *ETIL3*, *AP2*, *ANT*, and *SS2* emerged as core loci, suggesting their candidacy as genes involved in pineapple flower induction induced by ethephon (Figure 7A).

Flower induction is a complex network regulated by one or more pathways, and hormone signaling plays a crucial role in this context. Auxin, for example, is essential for initiating flower primordia in *Arabidopsis* [39]. Studies have shown that AUX/IAA interacts with ARFs and the activity of ARFs was inhibited. *LFY*, *ANT*, and *AIL6* are key regulators of flower induction that target *ARFs* for transcriptional activation. The dynamic expression of *LFY*, *ANT*, and *AIL6* strongly overlaps during flowering transition in *A. thaliana* [39]. In this study, we observed a decrease in the transcript level of *AUX/IAA25*, *ARF2*, *LFY*, *ANT*, and *AIL5* during flower induction (Figure 7B), which suggests a similar flowering pathway to that in *A. thaliana*. This implies that *AUX/IAA25* may interact with *ARF2* to regulate *LFY*, *ANT*, and *AIL5*, all of which are involved in flower induction induced by ethephon treatment. Another pathway involved in flower induction is the sugar signaling pathway. Clear evidence has shown that flowering time regulation is strongly influenced by the T6P signal, which integrates into the *miR156/SPL* node of the flower induction pathway [7,30]. *TPS1* increases the expression of *SPL15*, which in collaboration with *AP1* and *SOC1*, induces the expression of *miR172*. *MiR172*, in turn, inactivates the transcripts of *AP2*, ultimately leading to flower induction in *Arabidopsis thaliana* [47]. In our study, we observed an increase in the expression of *TPS9*, while *SPL14*, *SPL16*, and *AP2* showed decreased expression during flower induction (Figure 9), mirroring observations in *Arabidopsis thaliana*. This suggests that ethephon-induced pineapple flowering may involve the aging pathway of the flowering process. In conclusion, we propose that the sugar pathway and hormone pathway may work independently or in concert to mediate pineapple flowering induced by ethephon.

## 4. Materials and Methods

### 4.1. Plant Materials and Treatments

**Figure 10:**
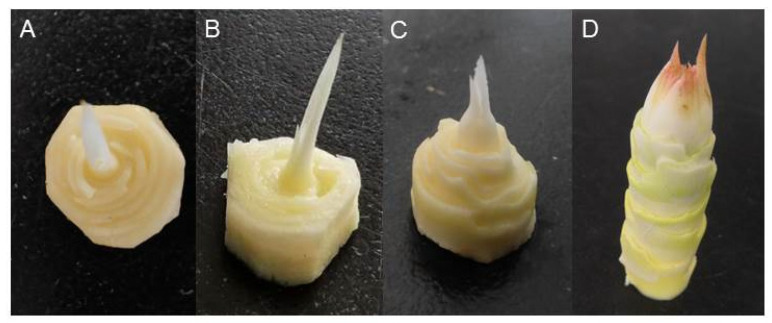
The morphological photographs of pineapple shoot apexes and inflorescences after ethephon treatment. The shoot apexes meristem or inflorescences were collected at different times with 400 mg·L^−1^ ethephon. (**A**) BL_0d, (**B**) BL_8d, (**C**) BL_18d, (**D**) BL_34d.

The pineapple cultivar ‘Comte de Paris’ was cultivated at the South Subtropical Crop Research Institute (21°10′02″ N; 110°16′34″ E), Zhanjiang, China. Homogeneous 13-month-old plants were used to induce flowering by ethephon. In October 2021, flowering was induced in each plant with 50 mL of 400 mg/L ethephon [26]. These samples were then collected at 0 d (shoot apexes meristem), 8 d (bract primordia formation stage), 18 d (flower primordia formation stage), and 34 d (stopped floret differentiation) and named BL_0d, BL_8d, BL_18d, and BL_34d, respectively. All samples were rapidly frozen in liquid nitrogen and stored at −80 °C. Thirty-five shoot apexes were mixed into a biological repetition in BL_0d and BL_8d, and 20 inflorescences were combined into a biological repetition in BL_18d and BL_34d (Figure 10) respectively, with three replicates for each time point.

### 4.2. Metabolomic Analysis

#### 4.2.1. Sample Preparation and LC–ESI-MS/MS System Analysis

The samples of BL_0d, BL_8d, BL_18d, and BL_34d were freeze-dried after being ground in a mortar with liquid nitrogen. Tissue samples (100 mg) were extracted overnight at 4 °C with 1.0 mL of 70% aqueous methanol containing 0.1 mg/L lidocaine as an internal standard. The extracts were then centrifuged at 10,000× *g* for 10 min, and the supernatant was collected and filtered (SCAA-104, 0.22-μm pore size; ANPEL, Shanghai, China) before LC–MS/MS analysis.

The compounds were analyzed using an LC-ESI-MS/MS system (UPLC, Shim-pack UFLC SHIMADZU CBM30A, Kyoto, Japan; MS/MS (Applied Biosystems 6500 QTRAP, Foster City, CA, USA). The high-performance LC analytical conditions were as follows: The Waters ACQUITY UPLC HSS T3 C18 column (2.1 mm × 100 mm, 1.8 μm) (Milford, MA, USA) was operated at 40 °C with a flow rate of 0.4 mL/min. The solvent system consisted of acidified water (0.04% acetic acid) and acidified acetonitrile (0.04% acetic acid). The gradient program was as follows: 95:5 *v*/*v* at 0 min, 5:95 *v*/*v* at 11.0 min; 5:95 *v*/*v* at 12.0 min, 95:5 *v*/*v* at 12.1 min, 95:5 *v*/*v* at 15.0 min. The effluent was connected to an ESI-triple quadrupole (QQQ)-linear ion trap (Q TRAP)–MS system. LIT and triple quadrupole (QQQ) scans were obtained from a Q TRAP, AB Sciex Q TRAP6500 System with an ESI-Turbo Ion-Spray interface. The Q TRAP was operated in positive ion mode, controlled by Analyst 1.6.1 software (AB Sciex, Toronto, ON, USA) with an ESI source temperature of 500 °C, ion spray voltage (IS) of 5500 V, and curtain gas (CUR) at 25 psi. The collision gas (CAD) was set to the highest level. QQQ scans were acquired as multiple reaction monitoring (MRM) experiments with the collision gas (nitrogen) set to medium. The declustering potential (DP) and collision energy (CE) for individual MRM transitions were performed with further DP and CE optimization.

#### 4.2.2. Differentially Accumulated Metabolites Analysis

The identified metabolites were searched against databases such as the MassBank, KNApSAcK, HMDB, MoTo DB, and METLIN [3]. Comparisons were made using *m*/*z* values, retention time (RT), and the fragmentation patterns with the standards. Metabolites with a *t*-test *p*-value of <0.05 and a variable importance (VIP) ≥ 1 were considered DAMs. Principal component analysis (PCA) and KEGG pathway enrichment were employed for metabolite analysis; three biological replicates were performed for each sample.

### 4.3. RNA-seq Analysis

#### 4.3.1. RNA Isolation and Library Construction

Total RNA was extracted from each sample using a Trizol reagent kit (Invitrogen, Carlsbad, CA, USA) following the manufacturer’s instructions. The RNA quality was assessed using an Agilent 2100 Bioanalyzer (Agilent Technologies, Palo Alto, CA, USA). Eukaryotic mRNA and prokaryotic mRNA were enriched using oligo (dT) beads and the Ribo-ZeroTM Magnetic Kit (Epicentre, Madison, WI, USA). The enriched mRNA was then fragmented and reverse transcribed into cDNA using random primers. Second-strand cDNA was synthesized using the first chain as a template. The cDNA fragments were purified using the QiaQuick PCR extraction kit (Qiagen, Venlo, The Netherlands), end repaired, and poly (A) added. The fragment products were selected by agarose gel electrophoresis and amplified by PCR. The library was sequenced using the Illumina HiSeq2500 platform by Gene Denovo Biotechnology Co. (Guangzhou, China).

#### 4.3.2. Differentially Expressed Genes (DEGs) Analysis

High-quality clean reads were obtained after filtering and removing the low-quality reads. The clean reads were aligned to the pineapple reference genome using HISAT2 v2.0.5. Differential expression analysis was performed using DESeq2 between two different groups based on the adjusted FPKM (fragments per kilobase of transcript per million mapped reads), and DEGs were filtered based on a false discovery rate (FDR) < 0.05 and an absolute fold change (|log2FC|) ≥ 2. All DEGs were mapped to terms of the Gene Ontology (GO) database (http://www.geneontology.org/ (accessed on 12 December 2021)) and KEGG database to significantly enrich GO and KEGG pathways.

### 4.4. Association Analysis between Transcriptome and Metabolome

To assess the correlation between genes and metabolites, Pearson correlation coefficients were calculated. The ‘cor’ program in the R project was utilized to select only differentially expressed genes (DEGs) and differentially accumulated metabolites (DAMs) with an absolute Pearson correlation coefficient ≥ 0.9. Subsequently, the metabolite–transcript network was visualized using the ‘igraph’ package in R.

### 4.5. qRT-PCR Verification of Gene Expression

To validate the gene expression results obtained from RNA-seq, qRT-PCR was performed on nine selected genes. Total RNA extraction and qRT-PCR procedures followed the methods described by Lin et al. [48]. qRT-PCR was conducted using a LightCycler 480 II instrument (Roche, Basel, Switzerland) with the SYBR Green qPCR Master Mix (Thermo Fisher Scientific, Waltham, MA, USA). Specific primers for each gene were designed using the Primer-BLAST program (https://www.ncbi.nlm.nih.gov/tools/primerblast/ (accessed on 15 April 2023)), and sequences for the genes are listed in Supplementary Table S4. Relative gene expression levels were calculated using the 2^−ΔΔCT^ method with β-actin as the internal reference gene for pineapple. Each sample underwent three biological replicates.

## 5. Conclusions

In our study, a total of 804 metabolites were isolated from pineapple shoot apex and inflorescence. These metabolites were classified into 24 categories. Notably, significant differences in the levels of 29, 31, and 46 metabolites were observed between BL_0d and BL_8d, BL_8d and BL_18d, and BL_18d and BL_34d, respectively. In addition to the metabolite analysis, we also identified differentially expressed genes (DEGs) in the pineapple samples. Specifically, we found 956 DEGs in the comparison between BL_0d and BL_8d, 1768 DEGs between BL_8d and BL_18d, and 4483 DEGs between BL_18d and BL_34d. These DEGs were significantly enriched in carbohydrate metabolism and hormone signaling pathways. Combining the metabolomics and transcriptomic analyses, several candidate genes, including *AGL9*, *ETIL3*, *AP2*, *ANT*, and *SS2*, were identified, suggesting their potential roles in pineapple flower induction. Moreover, the regulatory network of pineapple flower induction was constructed based on the pathway of *Arabidopsis thaliana*. Overall, this study provides valuable insights into the molecular and metabolic processes underlying pineapple flower induction by ethephon.

## Data Availability

Data is contained within the article and supplementary material.

## Supplementary Materials

The supporting information can be downloaded at: https://www.mdpi.com/article/10.3390/ijms242417133/s1.

## Abbreviations

BL	‘Comte de Paris’
qRT-PCR	Real-time quantitative reverse transcription polymerase chain reaction
