# Supplementary material for: Integrative Analysis of Metabolome and Transcriptome Provides Insights into the Mechanism of Flower Induction in Pineapple (Ananas comosus (L.) Merr.) by Ethephon
Source: Int J Mol Sci. 2023 Dec 5;24(24):17133. doi: 10.3390/ijms242417133 (PMC10742410; doi:10.3390/ijms242417133)
Supplement: Supplementary file 1 [file ijms-24-17133-s001.zip › illustration of supplementary figures and tables.pdf]

FigureS1. The correlation analyses between shoot apexes or inflorescences of pineapple. The blue was darker, indicating a stronger correlation.

FigureS2. Heatmaps of differentially accumulated metabolites (DAMs).

Figure S3. Heatmaps of DAMs in common.

FigureS4. The correlation analyses between shoot apexes or inflorescences of pineapple. The blue was darker, indicating a stronger correlation.

Table S1. The metabolites of shoot apexes or inflorescences.

Table S2. Summary of pineapple transcriptome.

Table S3. GO enrichment of DEGs.

Table S4. The primers of qRT-PCR.
