# Supplementary figures and images for: Integrative Analysis of Metabolome and Transcriptome Provides Insights into the Mechanism of Flower Induction in Pineapple (Ananas comosus (L.) Merr.) by Ethephon
Source: Int J Mol Sci. 2023 Dec 5;24(24):17133. doi: 10.3390/ijms242417133 (PMC10742410; doi:10.3390/ijms242417133)

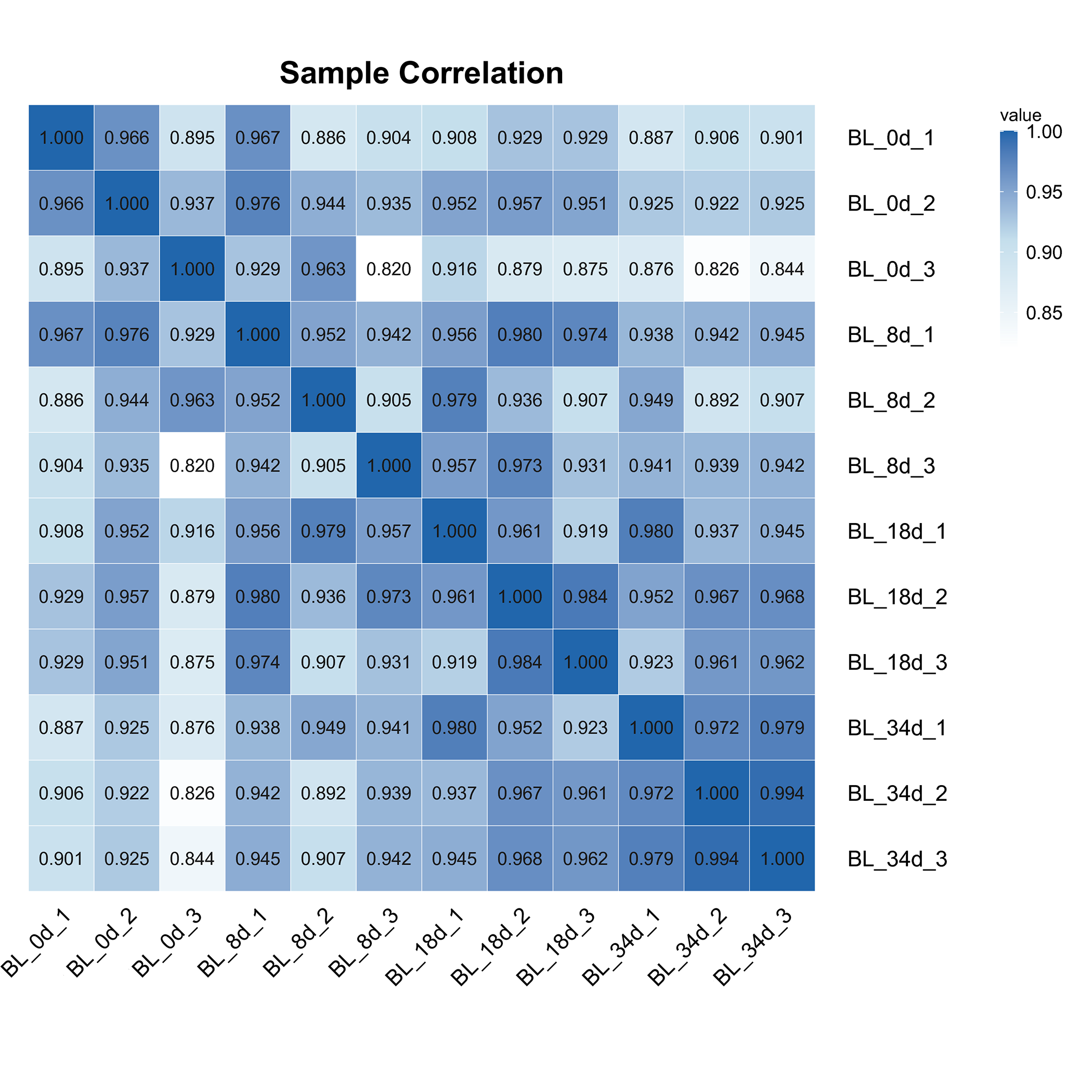

Supplement: Supplementary file 1 [file ijms-24-17133-s001.zip › Figure S1.png]

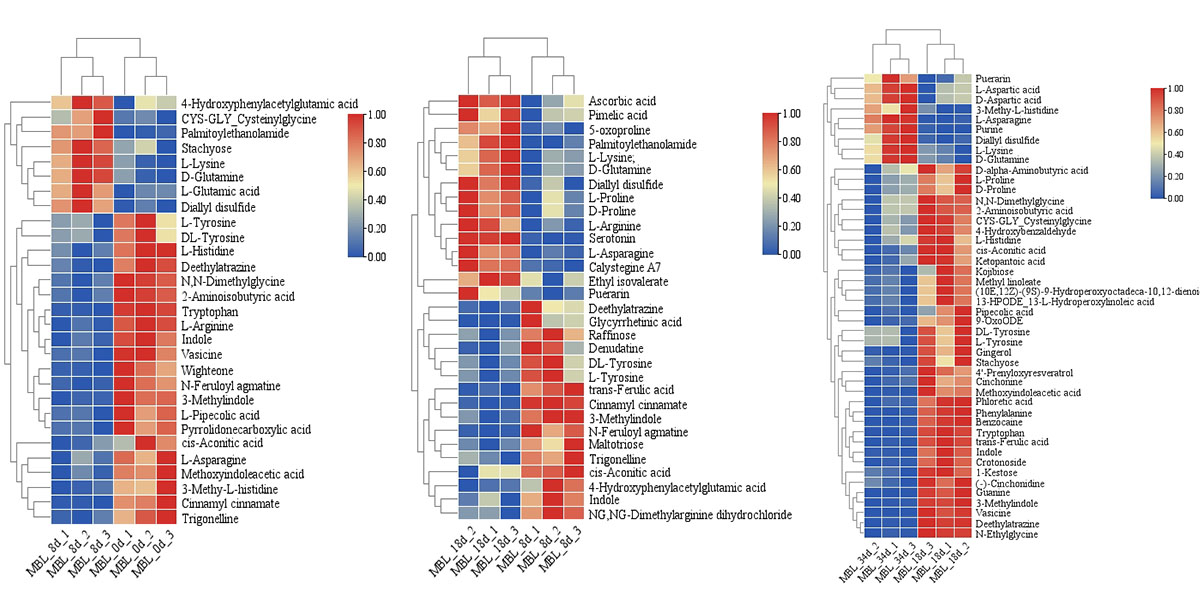

Supplement: Supplementary file 1 [file ijms-24-17133-s001.zip › Figure S2.jpg]

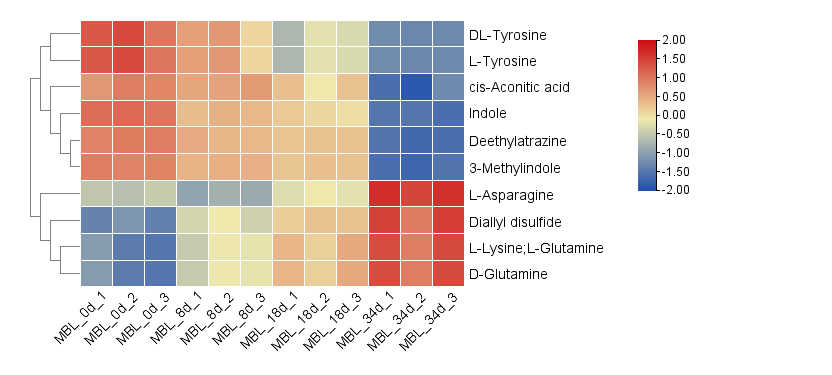

Supplement: Supplementary file 1 [file ijms-24-17133-s001.zip › Figure S3.png]

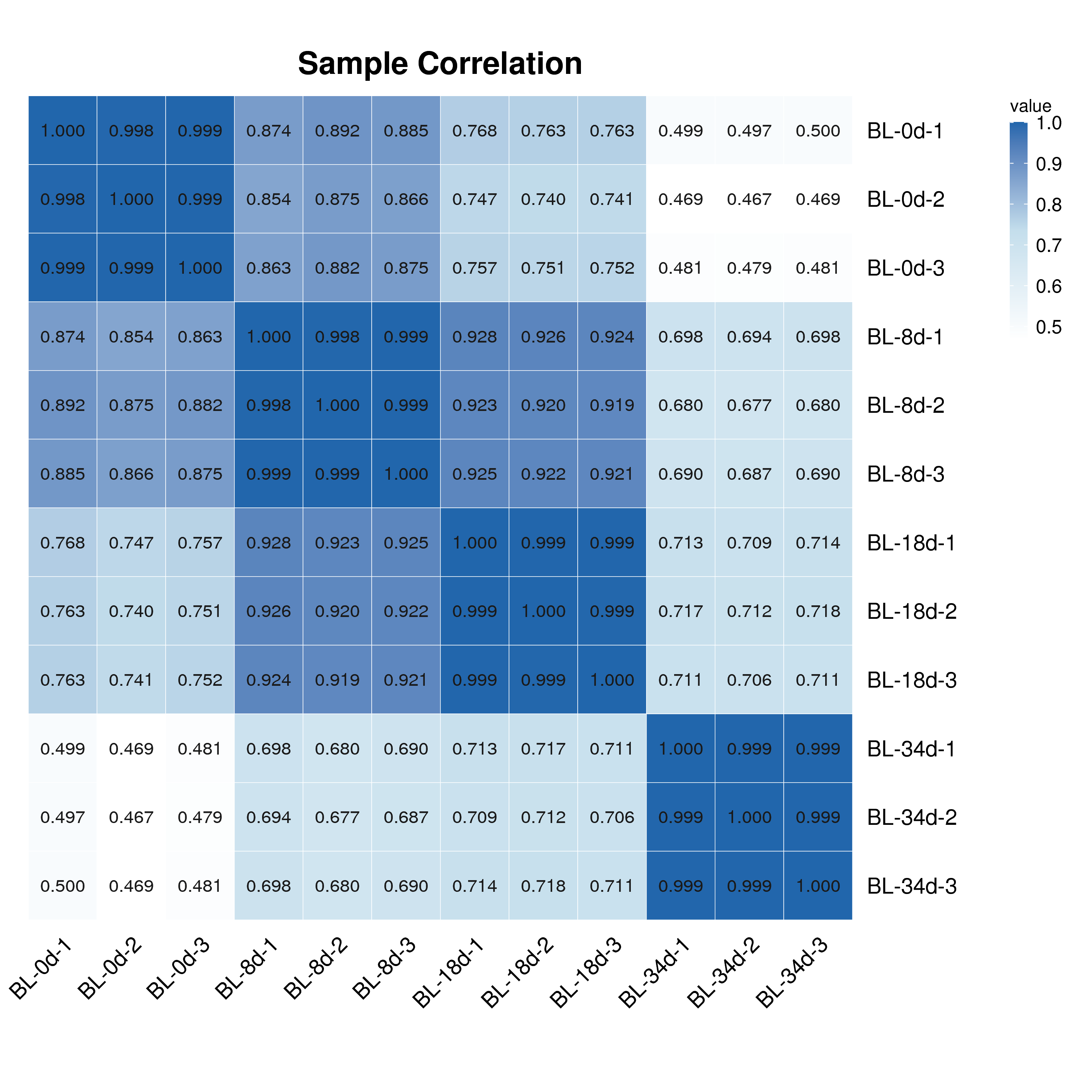

Supplement: Supplementary file 1 [file ijms-24-17133-s001.zip › Figure S4.png]
